# Supplementary material for: Testing the unitary theory of language lateralization using functional transcranial Doppler sonography in adults
Source: R Soc Open Sci. 2019 Mar 13;6(3):181801. doi: 10.1098/rsos.181801 (PMC6458414; doi:10.1098/rsos.181801)
Supplement: Supplementary Material [file rsos181801supp1.docx]

# Supplementary Materials

## Background

This project was preregistered on OSF under the title ‘CANDICE A2: Assessing reliability of language laterality measures using fTCD’.

The original protocol for project is here: <https://osf.io/tkpm2/registrations/>. The protocol was later amended, and a description of this can be found here: <https://osf.io/bjsv8/>.

The supplementary materials presented here detail the results of the analyses as they were planned in the original preregistered protocol, and explain the justification for amending the protocol.

## Change in Baseline Interval

## The baseline interval in the original protocol was -5 to 0 seconds. This was amended to -5 to 2 seconds, i.e. extending into the ‘Clear mind’ period. This decision was made after inspecting data from the current study and another ongoing study in our group, which revealed that the shorter baseline sometimes gave unstable values for the LI, as illustrated in Figure 1.

**Figure 1.** Illustration of the trend for larger SE values with the shorter baseline (i.e. values below the line), with a handful of points showing large differences in this direction. These data from all tasks x 30 subjects in current study.


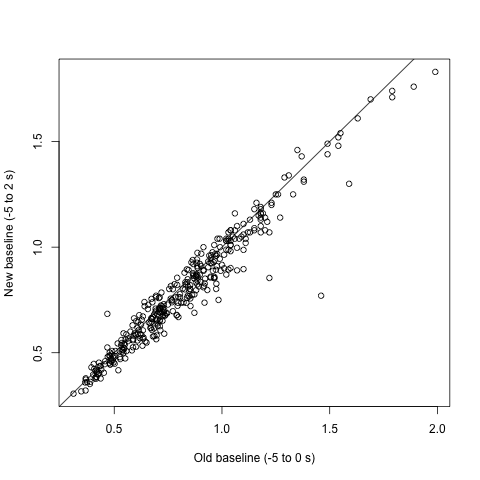


## Results from Preregistered Analysis

This results of the pre-registered analysis using the original 30 participants (23 R and L handers) are presented below. In this analysis, laterality index (LI) was defined in terms of peak absolute amplitude in the difference wave during the period of interest, and List Generation was used as the fixed path in test of bifactor model.

### Behavioural Results

We did not have specific predictions for the behavioural results, but present them here for completeness. For List Generation (A) and Sentence Generation (D), the number of words spoken per trial was recorded. The number of words spoken in both tasks and sessions were very similar: for task A, session 1, mean = 9.5, SD = 0.42, session 2, mean = 9.6, SD = 0.31; for task D, session 1, mean = 9.3, SD = 1.26, session 2, mean = 9.5, SD = 1.32. A repeated measures ANOVA showed no significant effects of task (F(1,29) = 0.47, *p* = 0.497) on the number of words spoken, but the effect of session was near significance (F(1,29) = 4.18, *p* = 0.05). Trials where participants failed to respond, or responded too early were excluded from analysis: these constituted less than 0.1% of trials.

For decision making tasks (B, C, E and F), the accuracy and RT of each response, and the number of omitted responses, were recorded (Table 2). Note that for task F participants were required to wait until the end of the word sequence before responding, and had only a second to respond; this accounts for the fast reaction times and relatively high number of omitted responses in task F.

The Phonological Decision and Sentence Comprehension tasks (tasks B and E) showed evidence of practice effects, as both accuracy and reaction times improved, and the number of omitted responses fell from Session 1 to Session 2.

**Table 1** Behavioural data for tasks B, C, E and F. The table shows mean percentage accuracy and reaction times (with SD), and results of t-tests comparing Session 1 with Session 2 for each measure. The number of omitted responses is reported as a percentage of all events. B = Phonological Decision; C = Semantic Decision; E = Sentence Comprehension; F = Syntactic Decision.

| **Measure** | **Session** | **Task B** | **Task C** | **Task E** | **Task F** |
| --- | --- | --- | --- | --- | --- |
| Accuracy (%) | 1 | 91.7 (5.22) | 96.1 (2.56) | 93.3 (4.33) | 90.3 (8.59) |
|  | 2 | 93.1 (4.32) | 95.3 (2.58) | 94.3 (4.02) | 90 (7.95) |
|  | 1 vs 2 | t=-2.39, p=.023 | t=1.35, p=.188 | t=-1.52, p=.140 | t=-0.14, p=.886 |
| Reaction times (s) | 1 | 1.62 (0.21) | 1.13 (0.2) | 2.16 (0.11) | 0.32 (0.07) |
|  | 2 | 1.46 (0.21) | 1.03 (0.19) | 2.1 (0.15) | 0.32 (0.07) |
|  | 1 vs 2 | t=8.16, p<.001 | t=4.35, p<.001 | t=2.78, p=.009 | t=-0.03, p=.978 |
| Omitted responses (%) | 1 | 2.15 | 0.81 | 2.67 | 3.93 |
|  | 2 | 0.74 | 0.48 | 1.67 | 4.44 |

## Lateralisation results

Four outlier LI values were excluded where the standard error across trials was above the upper cut-off. Four LI values were excluded because a subject had less than twelve useable trials for a given task in a given session. The remaining data for these participants were retained in the analysis.

Figure 1 shows density plots and Q-Q plots for each task, with results of the Shapiro Wilks normality tests. This shows that a number of the tasks showed non-normal distributions of LI values.


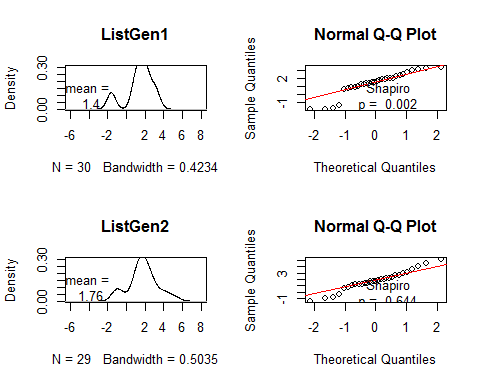

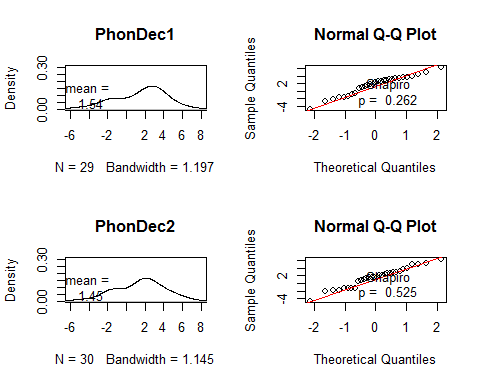

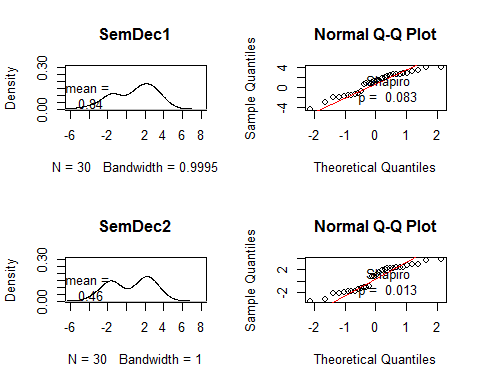

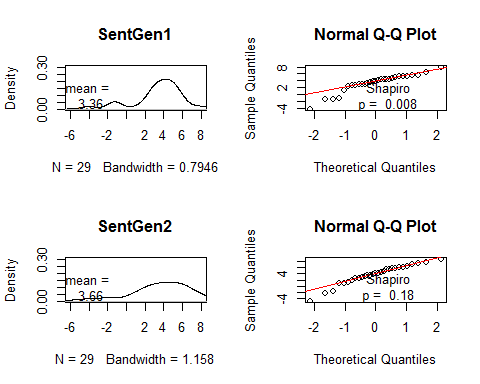

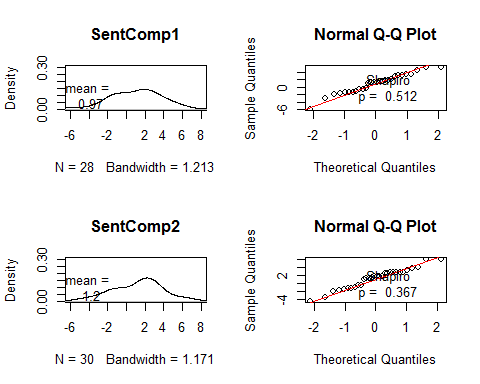

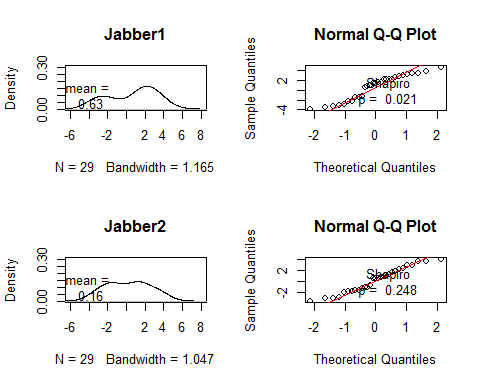


**Figure 1.** Density distributions and Q-Q plots for LI values from all tasks and sessions.

Figure 2 shows the distribution of LIs as a pirate plot (Phillips, 2017). Excluded datapoints are shown as red dots. Task D (Sentence Generation) showed the strongest left lateralisation. Shapiro-Wilks normality tests showed that LI values for four of 12 conditions were were non-normally distributed. One sample t-tests (testing for mean > 0) showed that all conditions were significantly left lateralised, except task C (Semantic Decision) at Session 2 (t (29) = 1.14, p=.133) and task F at both sessions (Syntactic Decision; Session 1: t (28) = 1.34, p = 0.095; Session 2: t (28) = 0.39, p = 0.351).


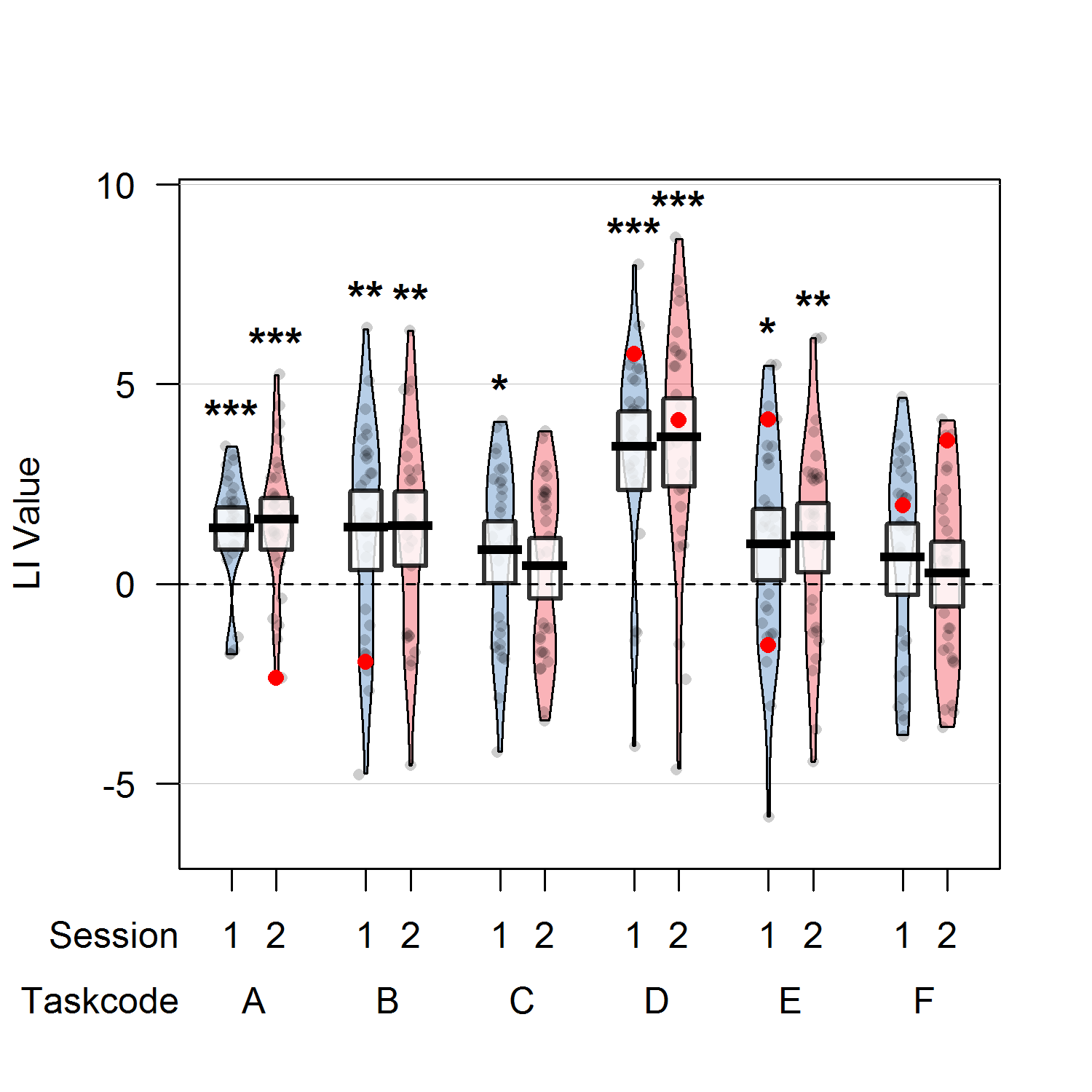


**Figure 2** Pirate plot of LI values for all tasks (A-F) and sessions (blue = Session1, pink = Session2). Excluded data-points are shown in red. Asterisks show results of Wilcoxon tests comparing the LI values of the group (omitting excluded data-points) to zero (* p<.05; ** p<.01; *** p<.001).

Figure 3 shows a correlation matrix of LI values for all tasks and sessions. Test-retest correlations varied between tasks. Task A (List Generation) had poor test-retest reliability (Pearson’s r = 0.22), and low correlations with other tasks. Test-retest reliability for other tasks ranged from r = 0.53 to 0.87. Tasks B, C, D and E were strongly intercorrelated. Task F (Syntactic Decision) had moderate test-retest reliability (r = 0.65) but relatively low correlations with other tasks.


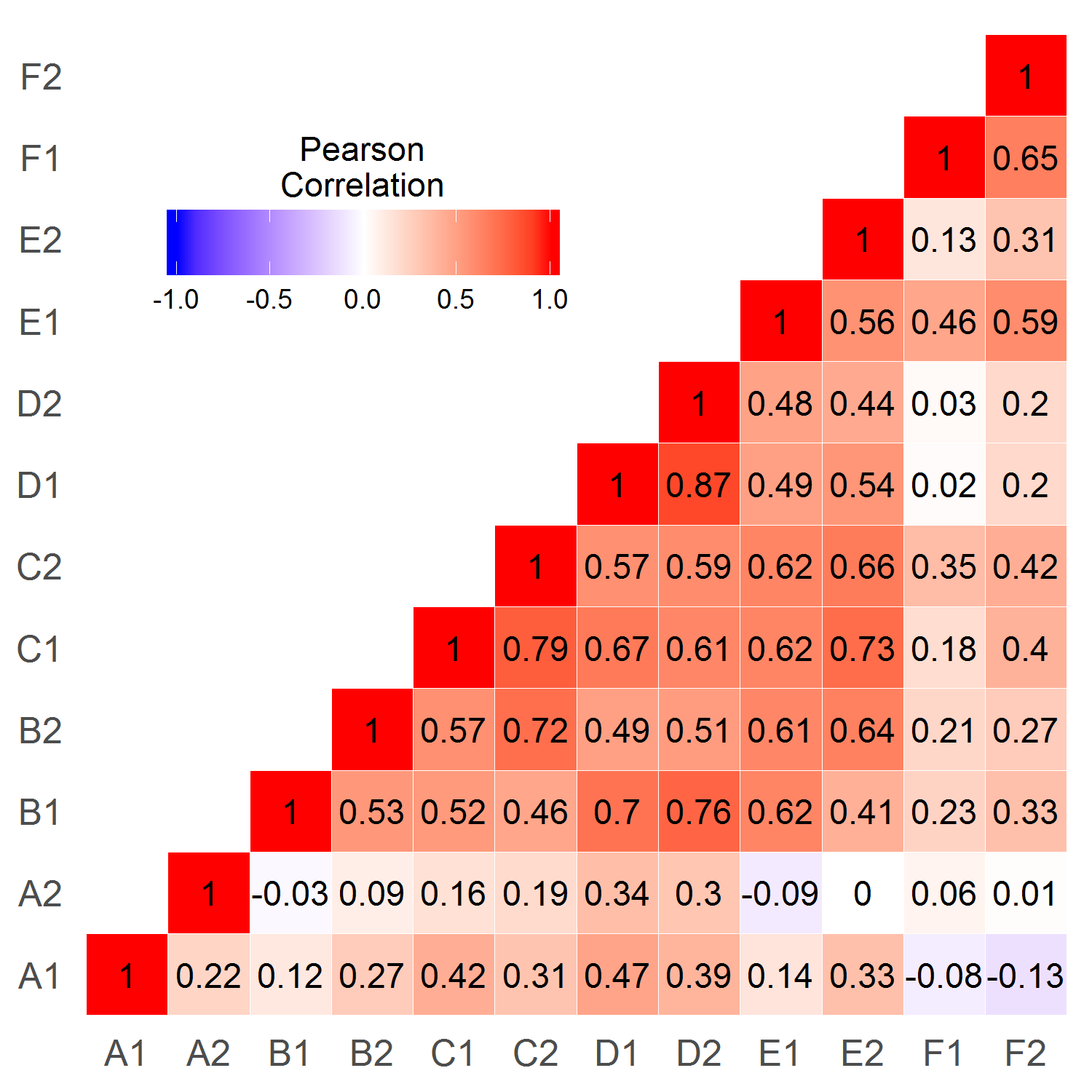


**Figure 3** Correlation matrix for LIs from the six language tasks given on two occasions.

##

## Structural Equation Modelling

The LI data were entered into the SEM analysis to test hypotheses about the group mean LI values and covariances in LI values across subjects. Table 1 summarises the SEM results.

### Step 1: Testing Stability of LI Values

As shown in Table 2, the fit of all the means-only models was very poor. This is to be expected, as these models ignore covariances, and, as indicated in Figure 3, there are substantial correlations both between and within tasks. Our interest at this point, however, is in the relative fit of different models of means, rather than overall model fit. The Fully Saturated model (with free means and variances) was compared to the Task Effect model, which fixed the means and variances for each task to be stable over sessions (i.e. A1 = A2, B1 = B2, etc.). The Task Effect model fit did not deteriorate significantly from that of the Fully Saturated model, supporting the hypothesis that LI means for each task were stable across sessions.

**Table 2** Model fit statistics from structural equation models and model comparisons. -2LogL = -2 log likelihoods; df = degrees of freedom; BIC = Bayesian Information Criterion; CFI = Comparative Fit Index; RMSEA = Root Mean Square Error of Approximation.

| **Model** | **Description** | **-2LogL** | **df** | **BIC** | **CFI** | **RMSEA** | **Chi Square test** | |
| --- | --- | --- | --- | --- | --- | --- | --- | --- |
|  |  |  |  |  |  |  | **Compared to** | **p** |
| Fully Saturated Model | Free means and variances | 1570.3 | 328 | 454.7 | NA | NA | - | NA |
| Task Effect Model | Stable means and variances | 1573.8 | 340 | 417.4 | 0.043 | 0.285 | Fully Saturated Model | 0.99 |
| Population Bias Model | Equal means and variances | 1659.1 | 350 | 468.6 | -0.337 | 0.317 | Task Effect Model | <.001 |
| Dorsal Stream Model | Means for tasks  AB > DEF > C | 1633.1 | 346 | 456.3 | -0.226 | 0.311 | Task Effect Model | <.001 |
| Lexical Retrieval Model | Means for tasks  BD > ACF | 1611.8 | 346 | 435 | -0.119 | 0.297 | Task Effect Model | <.001 |
| Person Effect Model | Covariances have one factor structure | 1422.2 | 334 | 286.2 | 0.777 | 0.143 | Task Effect Model | <.001 |
| Task x Person Effect Model | Covariances have bifactor structure | 1388.4 | 329 | 269.4 | 0.922 | 0.087 | Person Effect Model | <.001 |

### Step 2: Testing Models of Means

To demonstrate whether LI means differed between tasks, the Task Effect model (with different means for each task) was compared to the Population Bias model (with means fixed to be the same for all tasks). This may be seen as a null hypothesis that treats all tasks as equivalent measures of laterality. The Population Bias model gave significantly worse fit (see Table 2), supporting the hypothesis that LI means differed between tasks.

Two further models were compared to the Task Effect model. The Dorsal Stream model categorised the language tasks according to the involvement of the dorsal or ventral stream. Tasks A and B were categorised as involving strong dorsal stream activity, task C as strong ventral stream activity, and tasks D, E and F as intermediate (hence, means for AB > DEF > C). This model gave significantly poorer fit than the Task Effect model – as is evident from Figure 2, which shows relatively weak lateralisation for tasks A and B compared to task D. The Lexical Retrieval model did not fare any better. This categorised tasks B and D as involving strong lexical retrieval, whereas tasks A, C and F did not involve lexical retrieval, and task E was difficult to classify and so was considered as independent of the other measures (BD > ACF). Again, this model gave a worse fit than the Task Effect model, indicating that, while laterality varied between tasks, it did not fit the either of the predicted patterns. Note, however, that the pre-registered tests specified for both theories have some limitations, as discussed further below.

### Step 3: Testing Models of Covariances

At Step 3 we tested whether the covariances between tasks had a single factor structure (Person Effect model) or a bifactor structure (Task by Person Effect model). Not surprisingly, given the strong correlations in Figure 3, both within and across tasks, the Person Effect model gave substantially better fit than the Task Effect model (see Table 2); nevertheless, the overall fit of this model was poor. The Task by Person Effect model gave a significantly improved fit. A plot of the two factors is shown in Figure 4. Note that, although the model fit is not affected by task selection, the factor scores depend on which task has fixed paths to the factors. The paths for the case when List Generation is fixed are shown in Table 3.


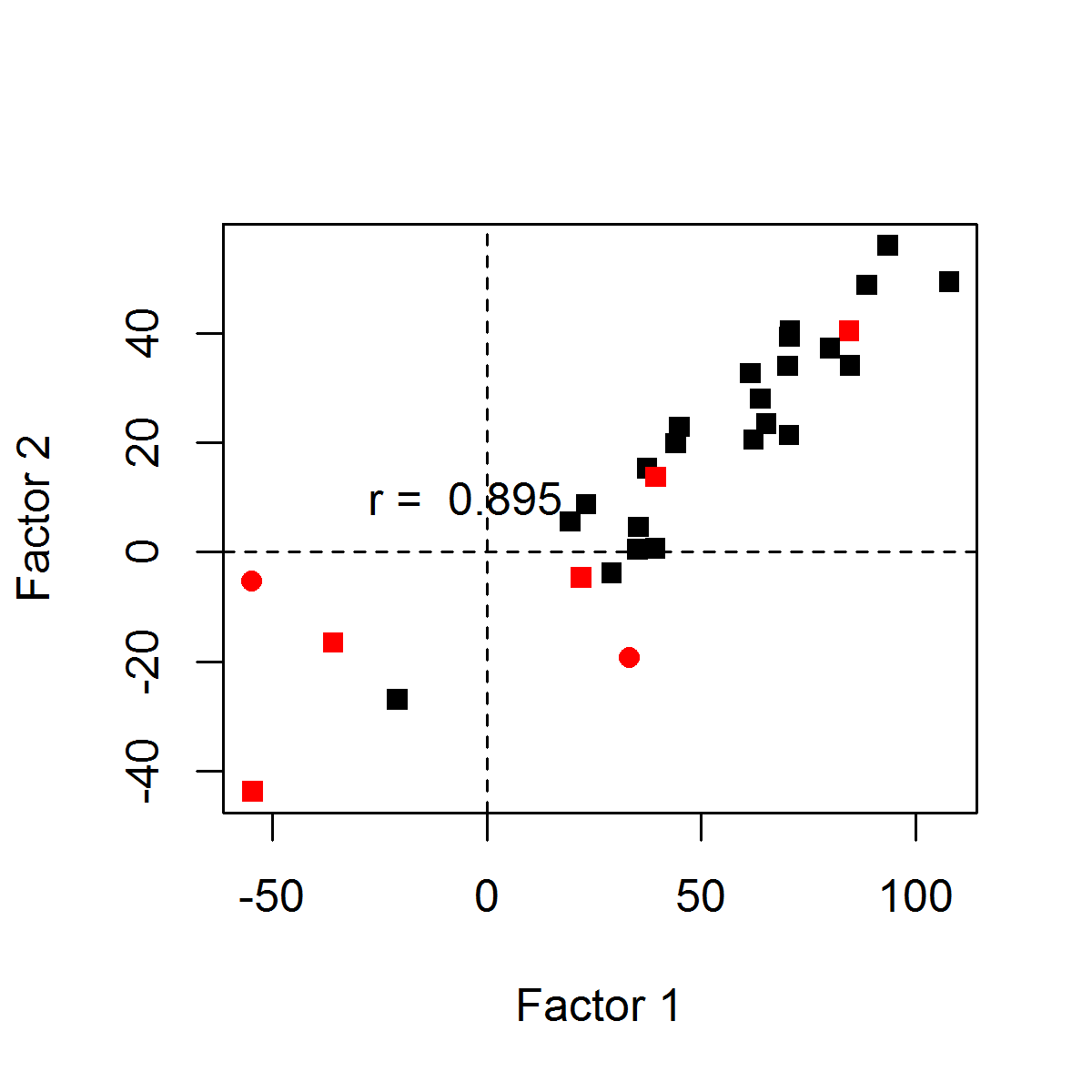


**Figure 4** Correlation between two factors from the bifactor (Task by Person Effect) model, with left-handers shown in red, and bivariate outliers as circles.

**Table 3** Path weightings (and 95% confidence intervals) from each latent factor (Factor 1 and Factor 2) to each task (A to F) from the winning bifactor model.

| **Task** | **Factor 1** | | **Factor 2** | |
| --- | --- | --- | --- | --- |
|  | **Path** | **95% CI** | **Path** | **95% CI** |
| A: List Generation | 1.00 | fixed | 0.00 | fixed |
| B: Phonological Decision | 2.25 | -0.09 to 4.59 | 1.23 | 0.14 to 2.32 |
| C: Semantic Decision | 1.79 | -0.46 to 4.04 | 1.48 | 0.64 to 2.31 |
| D: Sentence Generation | 4.07 | 1.3 to 6.84 | 0.73 | -0.88 to 2.35 |
| E: Sentence Comprehension | 1.47 | -1.07 to 4.01 | 1.75 | 0.89 to 2.6 |
| F: Syntactic Decision | -0.26 | -2.25 to 1.73 | 1.43 | 0.7 to 2.16 |

To test the robustness of the factor structure, a drop-one analysis was conducted, by re-running the models with 29 of the 30 participants. This showed that the bifactor model gave better fit than the single factor model on 29 of 30 runs.

Nevertheless, it is clear from Figure 4 that the two factors are highly intercorrelated, and the impression is that the factor solution is heavily affected by some influential cases. Cook’s distance identified two bivariate outliers, marked with circles in Figure 4: both outliers were left-handers.

### Exploratory analysis

Although the bifactor model gave a better fit than the single factor model, the fit statistics were relatively poor, and the drop-one analysis raised serious questions about the robustness of the finding, by indicating that removal of one single participant could remove support for the bifactor model. We therefore did additional analyses as follows:

1. First, we tackled the non-normality of the LI distributions by substituting a method of computing LI based on averaging the difference wave within the POI, rather than taking peaks. This considerably improved the normality of the data, but had little impact on the results of the SEM. The correlation between the two methods for LI estimation was very high.
2. Next, we changed the fixed path in the analysis. Rather than using List Generation, which had low reliability and weak relationships with other tasks, we used Sentence Generation, which was the most strongly lateralized measure. The revealed that selection of the fixed path variable has no effect on the model fit statistics, though it does give a different pattern of factor loadings.
3. We identified the participant that had a large influence on the drop-one analysis, and noted this was a left-hander. When the model was re-run with the 23 right-handers only, support for the bifactor model was lost.

On the basis of these findings, we concluded that the primary factor influencing the results was the composition of the sample rather than the specifics of how the LI was derived or how the analysis was conducted. In particular, the question arose of whether different models might apply to left- and right-handers. Accordingly, we decided to recruit more right-handers to test whether the bifactor model would still be superior if we had a sample of 30 individuals consisting only of right-handers. A revised protocol was pre-registered at this point, and results from the revised sample are reported in the main text.

A script is provided that allows readers to run the analysis to explore the impact of analytic decisions on the outcome, by varying the sample, inclusion/exclusion of left-handers, the method of computing the laterality index, and the fixed path. (<https://osf.io/q8zka/>).

### SEM analysis excluding List Generation

The List Generation task had low test-retest reliability and correlated poorly with other measures. In response to a reviewer’s concern, we re-ran the SEM analyses with five tasks only, excluding the list generation task. Tables 4 and 5 (directly comparable with Tables 2 and 3 in the manuscript) show that this did not affect the pattern of the results. Hence, we can conclude that inclusion of list generation did not drive the findings presented in the manuscript.

**Table 4** Model fit statistics from structural equation models and model comparisons, excluding List Generation (task A). -2LogL = -2 log likelihoods; df = degrees of freedom; BIC = Bayesian Information Criterion; CFI = Comparative Fit Index; RMSEA = Root Mean Square Error of Approximation.

| **Model** | **Description** | **-2LogL** | **df** | **BIC** | **CFI** | **RMSEA** | **Chi Square test** | |
| --- | --- | --- | --- | --- | --- | --- | --- | --- |
|  |  |  |  |  |  |  | **Compared to** | **p** |
| Fully Saturated Model | Free means and variances | 1367.6 | 342 | 132.7 | NA | NA | - | NA |
| Task Effect Model | Stable means and variances | 1372.3 | 352 | 101.3 | 0.022 | 0.338 | Fully Saturated Model | 0.909 |
| Population Bias Model | Equal means and variances | 1473.0 | 360 | 173.1 | -0.368 | 0.373 | Task Effect Model | <.001 |
| Dorsal Stream Model | Means for tasks  AB > DEF > C | 1440.9 | 356 | 155.4 | -0.25 | 0.369 | Task Effect Model | <.001 |
| Lexical Retrieval Model | Means for tasks  BD > ACF | 1400.9 | 356 | 115.4 | -0.081 | 0.343 | Task Effect Model | <.001 |
| Person Effect Model | Covariances have one factor structure | 1173.9 | 347 | -79.1 | 0.837 | 0.144 | Task Effect Model | <.001 |
| Task x Person Effect Model | Covariances have bifactor structure | 1135.8 | 343 | -102.7 | 0.981 | 0.051 | Person Effect Model | <.001 |

**Table 3** Path weightings (and 95% confidence intervals) from each latent factor (Factor 1 and Factor 2) to each task (B to F) from the winning bifactor model, excluding List Generation.

| **Task** | **Factor 1** | | **Factor 2** | |
| --- | --- | --- | --- | --- |
|  | **Path** | **95% CI** | **Path** | **95% CI** |
| B: Phonological Decision | 0.63 | 0.42 to 0.84 | 0.52 | 0.17 to 0.86 |
| C: Semantic Decision | 0.55 | 0.37 to 0.73 | 0.48 | 0.19 to 0.78 |
| D: Sentence Generation | 1.00 | Fixed | 0.00 | Fixed |
| E: Sentence Comprehension | 0.59 | 0.31 to 0.87 | 0.91 | 0.48 to 1.33 |
| F: Syntactic Decision | 0.14 | -0.14 to 0.41 | 1.20 | 0.82 to 1.58 |
